# Factors affecting women’s intention to use long acting and permanent contraceptive methods in Wolaita Zone, Southern Ethiopia: A cross-sectional study

**DOI:** 10.1186/1472-6874-14-109

**Published:** 2014-09-12

**Authors:** Mengistu Meskele, Wubegzier Mekonnen

**Affiliations:** 1 School of Public Health, College of Health Sciences, Wolaita Sodo University, PO Box: 138, Wolaita Sodo, Ethiopia; 2 School of Public Health, College of Health Sciences, Addis Ababa University, PO Box: 138, Addis Ababa, Ethiopia

**Keywords:** LAPMs, Intention, Attitude, Myths, Ethiopia

## Abstract

**Background:**

The use of long acting and permanent contraceptive methods (LAPMs) has not kept step with that of short-acting methods such as oral pills and injectable in Africa. This study explores the association between women’s awareness, attitude and barriers with their intention to use LAPMs among users of short term methods, in Southern Ethiopia.

**Methods:**

A cross-sectional study design of mixed methods was conducted in the public health facilities of Wolaita zone, Southern Ethiopia, in January 2013. Women who were using short term contraceptive methods were the study population (n = 416). Moreover, 12 in-depth interviews were conducted among family planning providers and women who have been using short term methods. Data were entered into EPI Info version 3.5.3 and exported to SPSS version 16.0 for analysis. The odds ratios in the binary logistic regression model along with 95% confidence interval were used.

**Results:**

One hundred fifty six (38%) of women had the intention to use LAPMs while nearly half of them (n = 216) had a negative attitude to use such methods. Moreover, two-third of study participants (n = 276) held myths and misconceptions about such methods. The women who had a positive attitude were found to be 2.5 times more intention to use LAPMs compared to women who had a negative attitude (AOR =2. 47; 95% CI: 1.48- 4.11). Women who had no myths and misconceptions on LAPMs were found to be 1.7 times more intention to use LAPMs compared to women who had myths and misconceptions (AOR = 1.71; 95% CI: 1.08- 2.72). Likewise, women who attained secondary and higher level of education were found to be 2 and 2.8 times more intention to use LAPMs compared to women with no education, respectively (AOR = 2. 10; 95% CI: 1.11- 3.98) and AOR = 2. 80; 95% CI: 1.15- 6.77).

**Conclusions:**

Intention to use LAPMs was low and nearly half of women had a negative attitude to use such methods. Positive attitude, absence of myths and misconceptions on LAPMs and secondary and plus level of education predicts intention to use LAPMs. Educating communities to change the attitude, myths and misconceptions on LAPMs should be aggressively done.

## Background

More than 200 million women in the developing world want to avoid pregnancy but are not using modern methods of contraception [1]. If the more effective LAPMs have been used, the number of unintended births and induced abortions could have been substantively reduced to help families and countries to achieve their health goals [2]. Moreover, avoiding barriers to the use of contraceptives and enhancing the demand for family planning could avert 54 million unintended pregnancies, more than 79,000 maternal deaths and one million infant deaths each year [1].

Many sub-Saharan African women fear side effects and mention health concerns as primary reasons for their unwillingness to use family planning in the future. Large numbers of women have exceeded their required fertility, but do not use family planning methods. Moreover, they use less effective temporary contraceptive methods [3].

In spite of their high effectiveness, the use of provider-dependent LAPMs of contraception, including intrauterine devices, hormonal implants, female sterilization, and vasectomy has lagged behind [4]. These methods are between 3 and 60 times more effective than short acting methods during a year of typical use. Access prevents wider use of such methods in Africa [5]. Only 2.7 million women are currently using these methods in Sub-Saharan Africa [6]. Thus, the use of LAPMs has not kept pace with short-acting contraceptive methods [7].

Ethiopia ranked the 12^th^ and 2^nd^ most populous country in the World and Africa respectively. The total population of the country was 87.1 million, according to 2012 population and economic development data sheet [8]. The total fertility rate was 4.8 in 2011; but the modern contraceptive prevalence rate (CPR) among married Ethiopian women was 27% [9].

The utilization of contraceptive methods was totally dominated by the use of short-term methods such as pills and injectable in Ethiopia. The most widely used methods were injectable (21%) followed by implants (3.4%), pills (2.1%), female sterilization (0.5%), IUD (0.3%), and male condom (0.2%). Moreover, the discontinuation rate of short term contraceptives was higher than that of LAPMs. The discontinuation rate for all methods was 37%, the highest of which was observed for pills (70%), followed by male condom (62%). Thus, such discontinuation rate of short term contraceptive methods was inefficient and expensive to the family planning program and resulted in many unintended pregnancies. However, implants had a discontinuation rate of only 5% [9].

Wolaita Zone is one of the most densely populated parts of the country with a crude population density of 385 people per square kilometer (ppkm^2^). The highest and lowest population density was shown in Damot Gale and Humbo Woreda of the zone; with 781 ppkm^2^ and 168 ppkm^2^ respectively [10]. Thus, the provisions of the most effective contraceptive methods such as LAPMs are very essential in a densely populated area like this.

With due emphasis on respecting clients’ rights and supporting informed decision making, the family planning program should focus on highly effective contraceptive methods, with a particular emphasis on LAPMs [11]. Moreover, the Minister of Health of Ethiopia (MOH) reported that lack of family planning method mix is among the challenges to implement the Health Sector Development program (HSDP III) effectively. This gap is also true in Wolaita zone. Ethiopia also visualizes scaling up of the family planning program with focus on long-term options [12]. Thus; this study will contribute to fill the information gap to scale up the family planning program and services with the focus to identify the factors hindering short term family planning user women’s intention to use LAPMs.

Furthermore, among the factors affecting the utilization of LAPMs, societal and familial opposition, lack of information about family planning and no formal education were barriers to use these methods [3]. Based on the factors which associated with women’s intention to use LAPMs in the other studies, we also hypothesized that women who had a positive attitude have more intention to use LAPMs than those women who had a negative attitude. Moreover, women who had secondary and higher level of education have more intention to use LAPMs than those women who had no education.

## Methods

### Study area and setting

A facility based cross-sectional study design of mixed methods was used. The study was conducted in Wolaita zone, Southern Nations, Nationalities and Peoples Region (SNNPR) of Ethiopia, in January 2013. Based on the most recent census (2007), which was conducted by the Central Statistical Agency of Ethiopia, the projected total population of the zone was 1,750, 079 with 863,043 males and 887,036 females on July, 2012 [13]. The study population for the quantitative method included short term family planning clients of selected health centers in Wolaita zone. On the other hand, key informant in-depth interview was administered to short term family planning users and their service providers.

### Sample size and sampling procedure

The sample size was determined by using a formula for estimation of single population proportion with the assumption of 95% confidence level, the margin of error of 5% and the prevalence of a client’s intention to use LAPMs in the future (56.1%) – taken from a study of Ambo town, Ethiopia [Dashe Negewo: Assessment of factors affecting women’s intention to use long acting and permanent contraceptive methods among family planning clients in Ambo town, Oromia National Regional state, Ethiopia, unpublished/ in preparation]. After considering 10% non-response rate, the total sample size was estimated to be 416.

A total of 65 rural and 5 urban health centers (2 newly constructed) were available in Wolaita zone. Six health centers (three from urban and another three from rural areas) were selected. The three rural health centers were randomly selected while all of the three health centers in urban Wolaita were included. Samples were allocated to each health center based on sampling proportionate to the size of family planning clients’ flow, which was observed three months prior to the data collection period (October, November and December 2013). Each short term family planning user who was visiting selected health centers during the study period were interviewed until the allocated sample size to every facility was reached. Furthermore, a purposive sampling technique was used to assess a total of 12 in-depth interviews (six of the key informants were short term family planning users while the remaining six were family planning service providers in the study health facilities). The clients and providers who participated in the interview had different socio-demographic backgrounds. Four of the key informants were housewives by their occupation; one of them was a merchant while the other one was a civil servant. Similarly, two of the providers, who participated in the interview, were males and the rest four were females. Moreover, three of the female providers were midwives while the other one female had B.Sc. in clinical nursing. Among male providers one had B.Sc. in clinical nursing while the rest had a diploma in clinical nursing.

### Data collection

The quantitative data were collected by using structured interviewer administered questionnaires; whereas key informant in-depth interview guide were developed for the qualitative study. The structured questionnaire was developed in English by reviewing different literatures considering the local situation [9,14]. The questionnaire was then translated into the official language of the southern region (Amharic) and back translated to English by a different person to check its consistency. Data collection was conducted by six health workers (diploma holders) who were not working in selected health facilities to minimize interviewer bias. Also two other health professionals, who had B.Sc. (nursing and public health) were supervised the data collectors.

The key informant in-depth interviews were used to gather greater depth of information to explore and understand the opinions and perceptions of short term family planning users and their providers. It also helped to triangulate the finding of the quantitative study. Some of the important issues addressed were awareness, attitude, rumours and misconceptions regards LAPMs.

### Data quality management and entry

Before the actual data collection, the questionnaire was pre-tested on similar setting outside the study area. The data collectors and supervisors were trained for two days on principles, ethical considerations, procedures and meanings of the questions included in the questionnaire. The principal investigator closely monitored the data collection process. Completed questionnaires were checked for their consistency and completeness every day. The qualitative data were audio taped in addition to note taking. Probing questions were used to explore more information on the issue under caption.

The quantitative data were entered and cleaned using in EPI Info version 3.5.3. The data were then exported to SPSS version 16 for analysis. The prevalence of knowledge, attitude and intention to use LAPMs was measured. Multicolinarity was checked before including similar variables in the regression model. Furthermore, crude and adjusted odds ratio in binary logistic regression model along with their 95% confidence interval were used to measure the strength and significance of the association between the various independent variables and intention to use LAPMs. In the qualitative study the data was audio taped, transcribed, translated and coded with Open Code version 3.6 and inductive content analysis was applied.

### Measurements

Ten awareness questions (with ‘Yes/No’ answers) - most of them taken from Ethiopian Demographic Health Survey 2011 and other literatures were included to measure the knowledge of women on LAPMs [9,14]. Then, knowledge score was computed based on the ten awareness questions. The score is calculated by adding values given to each of the ten questions. Then, it was categorized as low, moderate and higher knowledge. Those who knew 0–3 correct answers from ten knowledge questions were categorized as “Low knowledge”, while those who knew 4–6 and 7–10 correct answers from ten knowledge questions were categorized as “Moderate knowledge” and “Higher knowledge” respectively.

Likewise, attitude questions which were found in a research conducted in Mekelle town, Tigray region, North, Ethiopia was used to measure the attitude of women. The questions were measured using a liket scale - whether the clients strongly disagreed, disagreed, not sure, agreed and strongly agreed towards each of the attitude questions towards LAPMs [14]. An attitude score with three categories (disagree, agree, not sure) was computed using the same procedure that we used to compute knowledge score. Whereas, to assess the association between the outcome variable of interest and attitude, the composite variable was dichotomized using the median of the ten attitude questions. Those who scored less than or equals to the median score (four) of the ten correct attitude questions were categorized as “Negative attitude” whereas those who scored more than the median score (four) of the ten correct attitude questions were categorized as “Positive attitude”.

Furthermore, women were asked whether they had myths and misconceptions regarding LAPMs. Yet again, for those women who answered affirmatively as they had myths and misconceptions on LAPMs, specific myths and misconceptions questions such as implant causes hypertension, implant moves freely in the body and lost at the time of removal, implant causes illness, IUCD causes illness, implant causes anemia, IUCD causes anemia, LAPMs cause infertility, what do you know other myths and misconceptions were also paused.

### Operational definitions

Long-acting and permanent methods of contraception (LAPMs): are defined as those methods that prevent pregnancy more than or equals to three years per application (Implants, IUCD, male and female sterilizations).

Short term users: those clients who have been using Depo Provera, Pills and condoms (male, female).

Intention to use LAPMs: women who were not using LAPMs at the time of the survey but wanted to use such methods in the future.

### Ethical considerations

Ethical clearance was obtained from the Research Ethics Committee of School of the Public Health, College of Health Science, Addis Ababa University. Moreover, informed verbal consent was obtained from each study participant woman and health service provider. In order to ensure confidentiality, the women’s names were not written on the questionnaire. The privacy of participants was maintained. As well, the objectives of the study were communicated to each individual participant.

## Results

Out of 416 women approached, 411 women were included in this study, which makes the response rate to be 98.8%. The majority of women, 249 (60.6%) were in the age group of 25–34 years with a mean age of 26.7 (SD =5. 1). Among the 411 women interviewed, 285 (69.3%) were from urban areas. Almost all 370 (90%) of women were members of Wolaita Ethnic groups. Two hundred fifty four (61.8%) were Protestants and 128 (31.1%) were orthodox Christians by religion. One hundred thirty five (32.8%) women did not attend formal education. Moreover, 208 (50.6%) women had a family size of 1–4 persons while 203 (49.4%) of them had a family size of greater than or equal to five persons in their families with a mean household size of 4.9 (SD = 1.9). Almost the majority of women 399 (97.1%) was married. Almost half of women, 208 (51.23%) were married when they were aged less than 18 years. The mean age at first marriage were 17.6 (SD = 2.47). Most women had jointly decided with their partners on the number of children they wanted to have 237 (57.7%); while 38 (9.2%) and 136 (33.1%) of women reported that the decisions were made by their husbands and themselves alone respectively (Table 1).

**Table 1:** Socio- demographic and reproductive health characteristics of short term contraceptive method user women in Wolaita zone, Southern Ethiopia, January, 2013

**Characteristics (n = 411)**	**Frequency**	**Percent**
Age		
15-24	126	30.7
25-34	249	60.6
34 and above	36	8.7
**Resident type**		
Rural	126	30.7
Urban	285	69.3
**Religion**		
Protestant	254	61.8
Orthodox	128	31.1
Others*	9	7.1
**Educational status**		
No education	135	32.8
Primary education	142	34.5
Secondary education	96	23.4
Higher education	38	9.3
**Household size**		
1-4	208	50.6
5 and above	203	49.4
**Total number of children(n = 411)**		
<2	204	49.6
3 and above	207	50.4
**Decide on the number of children**		
Husband	38	9.2
Myself	136	33.1
Together	237	57.7
**Intention to use LAPMs**		
Yes	156	38
No	255	62
**Method intended to use in the future(n = 156)**		
Implants	142	91
IUCD	9	5.8
Female sterilization	5	3.2

*Muslim, Catholic, Apostolic, Jehovah witness, Traditional.

One hundred sixteen (28.2%), 37 (9%), 222 (54%), 361 (87.8%) of women were heard about female sterilization, male sterilization, IUCD and implant respectively. On the other hand, based on the composite LAMPs knowledge score, 165 (40.2%), 160 (38.9%) and 86 (20.9%) of women had low, moderate and higher knowledge respectively (Table 2). The qualitative finding augmented that knowledge on IUCD was poor and fear to use female sterilization and vasectomy were even rampant. Besides, even those who knew such methods believed living in rural areas hinders their use as it prevents the type of workload which rural families had to carry out. Some key informants said that they didn’t have enough knowledge of such methods, although health extension workers strived to bring about a change in their awareness. The situation was explained by a 32 year old married woman as: “We *got health information from health extension workers. I am using Depo Provera. I used Depo Provera for 6–7 year interval between every birth. I used implant few years ago, but it was not comfortable for me. I heard about the loop, but I am living in a rural area and the work load I have prevented me to use it. So the loop is not the best method for me. I heard about female sterilization too, but not vasectomy. The reason for not using IUCD is because, I am not educated. Our community has no knowledge on loop and vasectomy. I tried to use the implants, but it has side effects”.* Most service *p*roviders also mentioned there was a lack of awareness about LAPMs in their community. They explained that the majority of women had awareness on implant and IUCD but had less awareness on vasectomy and female sterilization.

**Table 2:** Knowledge of short term contraceptive user women on LAPMs and its characteristics in Wolaita zone, SNNPR Ethiopia, January, 2013

**Characteristics (n = 411)**	**Frequency**	**Percent**
Female sterilization	116	28.2
Male sterilization	37	9
Implants (Implanon/Jadelle/ Norplant)	361	87.8
IUD	222	54
IUCD can prevent pregnancies for more than 10 years	221	53.8
After female sterilization pregnancy is not possible	119	29
The implant can prevent pregnancies for 3–5 years.	349	84.9
Vasectomy has no interference with sexual intercourse	41	10
Implants require a minor surgical procedure during insertion and removal	317	77.1
IUCD is not appropriate for female at high risk of getting STIs	114	27.7

### Attitude and intention of women to use LAPMs in Wolaita, Southern Ethiopia

More than half (n = 216) of the women had a negative attitudes towards LAPMs. Women were asked to reflect their opinion on certain attitude questions. Two hundred eighty three (68.9%) of them agreed that using implants needs proper diet; while a quarter of them (n = 102) believed insertion and removal of implants is highly painful. More than one-fifth (n = 86) felt insertion of intrauterine contraceptive device interfere with privacy. About 69 (16.8%) of women admitted that using IUCD restricts normal daily activities**.** Similarly, nearly one quarter (109) of women agreed that female sterilization is dangerous. A third of study women (n = 137) believed that the implant might freely move in the body other than the site of insertion and cause severe pain. When women were asked for their approval, 125 (30.4%) of them agreed that it is not good to use LAPMs. Fifty seven (13.9%) of women believed that discussing about LAPMs with their husband or friend is not necessary. Meanwhile, 8 (1.9%) women understood that using LAPMs could not prevent from having the large family size. Ninety eight (23.8%) of women agreed that operation of women for female sterilization is unacceptable (Table 3). The qualitative finding was also in line with the quantitative study. Clients and providers alike believed that most of the community has a poor attitude towards LAPMs. But very few clients and providers believed that the community had a good attitude which can be enhanced through intensive awareness creation. For instance, a 25 year old married client from an urban health center explained: *“I have a negative attitude on LAPMs. Four years ago, I heard some rumors that a woman in my neighborhood developed mental illness after the implant inserted. After I knew the woman’s mental health was affected, I had developed a bad attitude towards LAPMs. I need to know more about LAPMs. I need to know in detail if any person with a better knowledge on LAPMs be able to teach me.”*

**Table 3:** Attitude of short term contraceptive method user women to use LAPMS in Wolaita zone, Southern Ethiopia, January, 2013

**Characteristics (n = 411)**	**Disagree # (%)**	**Not sure # (%)**	**Agree # (%)**
Using implants needs proper diet	89 (21.6)	39 (9.5)	283 (68.9)
The insertion and removal of implants are highly painful	113 (27.5)	196 (47.7)	102 (24.8)
Insertion of IUCD causes loss of privacy	173 (42.1)	152 (37)	86 (20.9)
Using IUCD restricts normal routine activities	162 (39.4)	180 (43.8)	69 (16.8)
Operation for female sterilization is dangerous	115 (28)	187 (45.5)	109 (26.5)
Implant freely move in the body and cause severe pain	197 (47.9)	77 (18.8)	137 (33.3)
For me, it is not good to use LAPMs	231 (56.2)	55 (13.4)	125 (30.4)
Discussing LAPMs with husband (friend) are not necessary	334 (81.2)	20 (4.9)	57 (13.9)
LAPMs not preventing one from having large family size	356 (86.6)	47 (11.5)	8 (1.9)
Operation for female sterilization is unacceptable	153 (37.2)	160 (38.9)	98 (23.9)

Fifty (12.2%) of the short term contraceptive method user women, who had ever used LAPMs in Wolaita zone, were implant users. Other LAPMs were not used by them. On the other hand, 156 (38%) women had the intention to use any of the four LAPMs in the future. The Implant was still the method highly favored for future use 143 (91.7%) followed by IUCD 9 (5.7%) and female sterilization 5 (3.2%) (Table 1).

### Myths and misconceptions to use LAPMs in Southern Ethiopia

Two hundred seventy six (67.2%) of women had myths and misconception on LAPMs. One hundred ninety one (46.5%) of women perceived that implant would cause hypertension. Only 162 (39.4%) of women believed that the implant could move around freely in the body and get lost on the day of removal. One hundred eighty seven (45.5%) and 116 (28.2%) of women believed that implant and IUCD cause illness respectively. In addition, 147 (35.8%) and 97 (23.6%) perceived that implant and IUCD leads to anemia, respectively. Moreover, 32 (7.8%) of women perceived that implant might cause infertility. Thirty four, (8.3%) of short term contraceptive user women believed in other myths and misconceptions about LAPMs (Table 4).

**Table 4:** S**hort term contraceptive user women, who had myths and misconceptions about LAPMs in Wolaita zone, Southern Ethiopia, January, 2013**

**Characteristics (n = 411)**	**Frequency**	**Percent**
Women who think there are barriers to use LAPMs	265	64.5
Myths and misconception heard on LAPMs	276	67.2
The implant would cause hypertension	191	46.5
Implant moves freely in the body and lost at the time of removal	162	39.4
Implant causes illness	187	45.5
IUCD Causes illness	116	28.2
Implant causes anemia	147	35.8
IUCD causes anemia	97	23.6
LAPMs cause infertility	32	7.8
Others*	34	8.3

*LAPMs interfere with routine activities if one has work load, LAPMs lead to twin pregnancy, LAPMs cause pregnancy to occur out of the uterus, LAPMs could suck blood, to use LAPMs one needs to have a balanced diet.

The qualitative finding also strengthened most of the findings in the quantitative study. Key informants had also implicated implant’s body burning and movement characteristics. They also mentioned that implant causes weakness, itching and it is not suitable for a person who has a great work load. As the implant and sterilizations presumed to require major operations, they were believed to cause pain to clients. Key informants also restated that LAPMs might cause cancer and mental illness. Moreover, there was also a belief that health professionals bulged the uterus outward at the time of loop insertion. The vasectomy also believed to make males impotent. A health provider (*23* years old midwife) explained the misconception as follows: *“Some women reported that implant moves to the abdomen and it is not found in the insertion site. It moves to other body parts from the insertion site. It interferes with the routine activities. If users of implant developed any headache or abdominal cramp they associate the situation with family planning method use.”*

### Factors associated with women’s intention to use LAPMs in Wolaita Zone, Southern Ethiopia

After adjusting for socio-demographic, knowledge and reproductive health characteristics, the women who had a positive attitude were found to be 2.5 times more likely to have the intention to use LAPMs compared to women who had a negative attitude (AOR = 2. 47; 95% CI: 1.48-4.11). Moreover, women who had no myths and misconceptions on LAPMs were found to be 1.7 times more likely to have the intention to use LAPMs compared to those who had myths and misconceptions (AOR = 1.71; 95% CI: 1.08-2.72). Likewise, women who attained secondary and higher level of education were found to be 2 and 2.8 times more likely to have the intention to use LAPMs compared to women who had no education, respectively (AOR = 2. 10; 95% CI: 1.11-3.98) and AOR = 2. 80; 95% CI: 1.15-6.77) (Table 5).

**Table 5:** Factors associated with women’s intention to use LAPMs in Wolaita zone, Southern Ethiopia, January 2013

**Characteristics (n=411)**	**Intention to use LAPMs**		**COR (95% CI)**	**AOR (95% CI)**
	**Yes # (%)**	**No # (%)**		
**Age**				
15-24	59 (46.8)	67 (53.2)	1	SM
25-34	85 (34.1)	164 (65.9)	0.59 (0.38-0.91)*	0.86 (0.47-1.53)
35 and above	12 (33.3)	24 (66.7)	0.57 (0.26-1.23)	0.89 (0.34-2.31)
**Household**				
1-4	92 (44.2)	116 (55.8)	1	1
5 and above	64 (31.5)	139 (68.5)	0.58 (0.39-0.87)*	0.81 (0.47-1.41)
**Educational status**				
No education	40 (29.6)	95 (70.4)	1	1
Primary education	41 (28.9)	101 (71.1)	0.96 (0.57-1.62)	0.85 (0.48-1.48)
Secondary education	51 (53.1)	45 (46.9)	2.69 (1.58-4.64)*	2.10 (1.11-3.98)**
Higher education	24 (63.2)	14 (36.8)	4.07 (1.91-8.67)*	2.80 (1.15-6.77)**
**Decide on the number of children**				
Husband	8 (21.1)	30 (78.9)	1	1
Myself	44 (32.4)	92 (67.6)	1.79 (0.76-4.23)	1.53 (0.62-3.80)
Together	104 (43.9)	133 (56.1)	2.93 (1.29-6.67)*	2.22 (0.93-5.29)
**Knowledge score**				
Low knowledge	56 (33.9)	109 (66.1)	1	1
Moderate knowledge	59 (36.9)	101 (63.1)	1.37 (0.72-1.79)	0.75 (0.43-1.31)
Great knowledge	41 (47.2)	45 (52.3)	1.77 (1.04-3.02)*	0.74 (0.36-1.52)
**Attitude score (composite)**				
Negative attitude	62 (28.7)	154 (71.3)	1	1
Positive attitude	94 (48.2)	101 (51.8)	2.31 (1.54-3.47)*	2.47 (1.48-4.11)**
**Heard myths and misconceptions**				
Yes	95 (34.4)	181 (65.6)	1	1
No	61 (45.2)	74 (54.8)	1.57 (1.03-2.39)*	1.71 (1.08-2.72)**

*Statistically significance in COR: P-value < 0.05, **statistically significance in AOR.

P-value < 0.05.

## Discussion

This study identified 156 (38%) of short term contraceptive user women, who had the intention to use LAPMs in the future. This finding was less than a study conducted in Ambo town, Ethiopia, 2010, where 56.1% of 519 women who were using family planning methods had the intention to use LAPMs [Dashe Negewo: Assessment of factors affecting women’s intention to use long acting and permanent contraceptive methods among family planning clients in Ambo town, Oromia National Regional state, Ethiopia, unpublished/ in preparation]. The difference could be due to the disparities in the study settings. The low intention documented in this study could be due to the myths and misconceptions on LAPMs. Of all LAPMs, 143 (91.7%) of the study participants intended to use the implant, which could be attributed to a higher level of knowledge on implant 361 (87.8%). This finding is in line with the finding of Ethiopia Demographic and Health Survey 2011 where the majority of the women (67.8%) had better awareness on implant among LAPMs [9]. This might be attributed by the training, which has been given to primary health extension workers on Implanon insertion by the Minister of Health [11]. Moreover, the availability of a variety type of implants in terms of durations such as (Implanon, Jaddle, and Norplant) and the accessibility of the services at every level of health facilities. However, it is inconsistent with the Pakistani study, which was done in 20 selected cities among 4,062 currently non-pregnant and married women aged 15–49 years and their partners in the year 2010; which also showed a higher level of women intending to use female sterilization than implant. Female sterilization was also the most widely used LAPMs worldwide, accounting for approximately (20%) of all contraception, followed by IUD [15,16].

Knowledge on vasectomy (9%) and implants (87.8%) were the lowest and the highest respectively; which is in line with the findings of a study conducted by Mekonnen and Worku in Butajira, South Central Ethiopia, 2009, which has the sample size of 5746 married women aged 15–49, were interviewed. The Butajira study showed that vasectomy and implants were known by (8.2%) and (74.4%) of study participants, respectively. Moreover, it is similar to the findings of Ethiopia Demographic and Health Survey 2011, in which the prevalence of knowledge on vasectomy and implant were 11.2% and 67.8% respectively. However, knowledge on IUCD (54%) and tubaligation (28.2%) were higher than Butajira study, which documented the knowledge level of IUCD (3.1%) and tubaligation (19%). The difference could be due to the fact that the government is scaling up implant and IUCD training for HEWs [9,11,17].

In this study, more than half (n = 216) of the study participants had a negative attitude to use LAPMs. This finding is almost similar to the study conducted in Mekele, 2011, Northern Ethiopia, where 53.6% of 460 married women of reproductive age had a negative attitude towards the use of LAPMs [14]. Such a negative attitude could be due to the widespread existence of different myths and misconceptions about LAPMs in different Ethiopian communities.

Women who attained secondary and higher level of education were found to be 2 and 2.8 times more likely to have the intention to use LAPMs compared to women who had no education, respectively (AOR = 2. 10; 95% CI: 1.11-3.98) and AOR = 2. 80; 95% CI: 1.15-6.77). This study was consistent with the findings of similar studies in Pakistan, Rwanda and Mojo, Ethiopia [16,18,19]. In the Rwanda study, a total of 637 non-pregnant, porous- women in unions, ages 21 to 49, in five districts were interviewed in November 2009 and February 2010; whereas a total of 551 women aged 15 to 49 were included in the survey of Mojo town in June 2011. This could be due to the fact that educated women had more access to information from different sources, like school, leaflet, newspaper, media and internet. In contrast to this finding, a study conducted in Bale zone, Goba town, Ethiopia, among 734 married women of reproductive age in September 2009, showed that there was no statistical significance association between education and use of LAPMs [20].

On the other hand, women who had a positive attitude were found to be 2.5 times more likely to have the intention to use LAPMs compared to women who had a negative attitude (AOR = 2. 47; 95% CI: 1.48-4.11). This finding was consistent with a study in Ambo, Ethiopia, in which a total of 519 women aged 18–49 years were interviewed in public health facilities, in August and June, 2010 [Dashe Negewo: Assessment of factors affecting women’s intention to use long acting and permanent contraceptive methods among family planning clients in Ambo town, Oromia National Regional state, Ethiopia, unpublished/ in preparation]. This finding was also supported by the key informant interviews which state that lack of adequate awareness of long acting and permanent methods in the community due to the spread of various myths and misconceptions on such methods.

The prevalence of myths and misconception in this study was 67.2%. This finding is approximately similar to the study done in Ghana, where the district-level contraceptive prevalence rate and couple year protection data, comes from regional and district reporting through the Ghana health service in 2007, in which 63% of clients said that it was because of the rumors that clients might not choose IUD. Similarly, 39.4% of short term method user women reported that implant moves freely in the body and lost at the time of removal were also closely comparable to a Ghanaian study finding of 35%. On the other hand, this finding is higher than the facility based study conducted in East Shoa Zone, Batu, Ethiopia in May 2009 among 398 women aged 18–49 years showing only about a third had myths and misconception. This difference could be due to the study setting, duration and variations in the cultures of the Oromo and Wolaita ethnic groups living in Batu and Waliata areas respectively [21,22].

Moreover, women who had no myths and misconceptions on LAPMs were found to be 1.7 times more likely to have the intention to use LAPMs compared to those who had myths and misconceptions (AOR = 1.71; 95% CI: 1.08-2.72). This finding is consistent with the study in Pakistan, where some women believed and perceived that family planning can harm a woman’s womb and also modern method can be dangerous were less likely to use contraceptive methods in the future [16].

The study used a mix of quantitative and qualitative methods to triangulate findings which could be cited as strength. The study was also able to identify different barriers to the use of LAPMs. Temporal relationship between exposure and outcome variable could not be established as it is a cross-sectional study and the fact that it is an institutional study might undermine generalizing the result to the general populations. Although an equal number of health facilities were selected from urban and rural areas, relatively few women selected from rural area might be considered as another limitation.

## Conclusions

In this study, more than half of women had a negative attitude towards use of LAPMs. Moreover, women had low intention to use LAPMs in Wolaita zone. Secondary and higher level of education, positive attitude to use such methods and having no myths and misconceptions were significant predictors of intention to use LAPMs among short term method user women. Furthermore, the study documented different myths and misconceptions as barriers to use LAPMs. Front line primary health workers and programmers need to scale-up awareness creation programs to change the attitude, myths and misconceptions of clients on LAPMs.

## Competing interests

The authors declare that they have no competing interests.

## Author’s information

MM is MPH in reproductive and family health, lecturer in the School of Public Health, College of Health Science, Wolaita Sodo University, Wolaita Sodo, Ethiopia. WM is Ph.D. in Public Health, Assistant Professor at School of Public Health, College of Health Science, Addis Ababa University, Addis Ababa, Ethiopia.

## Authors’ contributions

MM has taken a principal role in the conception of ideas, developing methodologies and writing the article. WM guided in the conception and design and involved in the analysis and interpretation of findings. Both authors read and approved the final version of the manuscript.

## Pre-publication history

The pre-publication history for this paper can be accessed here:

http://www.biomedcentral.com/1472-6874/14/109/prepub
